# PCR profiling of tumor-associated microorganisms in tissue of primary epithelial malignant tumors of colorectal origin: associations with key clinicopathological characteristics

**DOI:** 10.3389/fcimb.2026.1793881

**Published:** 2026-05-28

**Authors:** Nikolay K. Shakhpazyan, Liudmila M. Mikhaleva, Nikolay K. Sadykhov, Konstantin Y. Midiber, Roman V. Afanasev, Zarina V. Gioeva, Alexander I. Mikhalev, Arkady L. Bedzhanyan

**Affiliations:** 1 Avtsyn Research Institute of Human Morphology, Russian National Research Center of Surgery named after B.V. Petrovsky, Moscow, Russia; 2 Institute of Medicine, Peoples’ Friendship University of Russia named after Patrice Lumumba, Moscow, Russia; 3 Department of Hospital Surgery No. 2, Pirogov Russian National Research Medical University, Moscow, Russia; 4 Department of Abdominal Surgery and Oncology II (Coloproctology and Uro‐Gynecology), Russian National Research Center of Surgery named after B.V. Petrovsky, Moscow, Russia

**Keywords:** bacterial load, colorectal neoplasms, neoplasm grading, real-time polymerase chain reaction, *Ruminococcus*

## Abstract

**Introduction:**

Colorectal cancer is one of the most common malignancies worldwide, and microbiome research has strong potential to advance understanding of tumor biology and to support biomarker development and microbiome-targeted interventions. Most colorectal cancer microbiome studies rely on stool or mucosal sampling, but routine pathology archives contain abundant formalin-fixed, paraffin-embedded primary tumor tissue that could enable scalable assessment of tumor-associated microorganisms.

**Methods:**

We profiled tumor-associated microorganisms in primary colorectal adenocarcinoma specimens from 192 patients using a targeted quantitative PCR panel and evaluated associations with clinicopathological variables (tumor differentiation grade, primary tumor T category, and disease stage) using non-parametric tests, ordinal regression, regularized logistic regression, and within-panel profile ordination with permutation-based inference.

**Results:**

The most consistent signals were linked to tumor differentiation rather than stage. Tumors in the G2–G3 versus G1 comparison showed higher total bacterial load and a higher detection frequency of *Ruminococcus* spp., with both associations remaining significant after false discovery rate control; regression analyses corroborated these findings. A regularized logistic model achieved moderate discrimination for high-grade (G2-G3) disease (mean area under the receiver operating characteristic curve ≈0.71) and yielded a compact signature dominated by total bacterial load and detection of *Ruminococcus* spp., with additional contributions from low-frequency taxa that warrant cautious interpretation. In contrast, models targeting primary tumor T category, invasiveness (T1–T2 vs T3–T4), or overall stage showed low discriminative performance (area under the curve ≤0.59), and within-panel profile distances did not reveal robust global separation of groups.

**Conclusions:**

Targeted quantitative PCR profiling of archived primary tumor tissue identifies reproducible microbiome signals that track tumor differentiation grade more strongly than stage, suggesting that tissue bacterial burden and selected taxa may reflect microenvironmental features associated with the G2–G3 versus G1 contrast. Broader tumor microbiome profiling should be required to capture diversity and refine clinically informative signatures.

## 1 Introduction

Colorectal cancer (CRC) is one of the most prevalent problems in oncology. According to 2022 estimates, the global incidence of CRC was 1, 926, 425 cases per year across all sexes and age groups, accounting for approximately 9.6% of all new cancer cases. In terms of mortality, CRC ranks second among malignant neoplasms (≈9.3% of cancer-related deaths), corresponding to 904, 019 deaths worldwide. The 5-year prevalence (the number of individuals living within 5 years after diagnosis) is 73.2 per 100, 000 population (Bray et al., 2024). According to the World Health Organization (WHO), the burden of CRC is expected to increase by 2040 to ~3.2 million new cases per year and ~1.6 million deaths per year. This increase is thought to be driven by population ageing, population growth, and the prevalence of risk factors (Colorectal cancer, n.d.).

Thus, CRC represents a major healthcare challenge and an age-associated disease, linked to other common age-related conditions (cardiovascular diseases, metabolic syndrome) (Zeng et al., 2022; Han et al., 2024; Zhang et al., 2024). This underscores the need to develop preventive strategies and early detection approaches at the level of global public health programs aimed at achieving high levels of healthy longevity in the population.

CRC is directly linked to the most biomass-rich and biodiverse microbiome in the human body—the colonic (large-intestinal) microbiome. Accumulating evidence indicates that the gut microbiome is a significant and modifiable factor influencing colorectal carcinogenesis and treatment outcomes. Microbiome signatures are being explored as biomarkers for disease monitoring, and interventions targeting microbiome composition (including fecal microbiota transplantation, probiotics, and prebiotics) are being actively investigated in both preclinical and clinical settings (Liu et al., 2023b; Chen et al., 2025). Accordingly, microbiome research in CRC is among the most promising in terms of translational potential and offers hope for the development of robust protective antitumor strategies as tools for prevention and treatment of this disease.

Microbiome studies based on formalin-fixed, paraffin-embedded (FFPE) tissues remain relatively limited, despite the major practical value of pathology archives as a source of clinically annotated material. The main methodological challenges include formalin-related DNA damage and fragmentation, low microbial biomass, high background of host DNA, and the risk of reagent- or environment-derived contamination, all of which complicate accurate characterization of tissue-associated microorganisms. At the same time, recent reviews and experimental studies indicate that microbiome analysis from FFPE material is feasible, including in colorectal tissue, and may reveal biologically informative differences between tumor and non-tumor samples when these technical constraints are taken into account (Cruz-Flores et al., 2022).

Against this background, a targeted qPCR strategy may represent a pragmatic approach for FFPE-based studies when the objective is not broad taxonomic discovery, but quantitative evaluation of a predefined set of clinically relevant microorganisms. Compared with broader sequencing-based approaches such as 16S rRNA amplicon profiling, targeted qPCR does not provide comprehensive community-level characterization; however, it is better aligned with low-input and degraded archival material and with hypothesis-driven analysis of specific microbial targets in routine clinical samples. This is particularly relevant in FFPE colorectal tissue, where previous work has shown that qPCR-based bacterial marker analysis is feasible, whereas 16S amplicon sequencing remains technically more challenging and especially sensitive to contamination-related and host-DNA-related interference (Lam et al., 2021).

The aim of our study was to use a targeted quantitative PCR (qPCR) panel of key gut microbiota microorganisms to assess the presence of microorganisms in histological specimens of primary colorectal tumors and to evaluate their associations with key clinicopathological characteristics of CRC.

## 2 Materials and methods

### 2.1 Characteristics of patients with CRC

The study included 192 patients with CRC (108 men and 84 women) aged 32 to 95 years (median age 67 years [61–73]). Colon cancer was diagnosed in 87 patients, sigmoid colon cancer in 33, cancer of the rectosigmoid junction in 19, and rectal cancer in 52; in one case, the tumor had a multifocal localization (adenocarcinoma of the colon and sigmoid colon).

In all enrolled patients, the histological tumor type was adenocarcinoma. Tumor grade was assessed using a three-tier system: well-differentiated (G1) in 108 patients, moderately differentiated (G2) in 74, and poorly differentiated (G3) in 10.

Among the 192 patients, TNM stage grouping was performed according to the American Joint Committee on Cancer (AJCC) TNM Staging System (8th ed., 2017 for colon cancer; 8th ed., 2017 for rectal cancer), with the following distribution: stage I—25 (13.0%), stage II—72 (37.5%), stage III—53 (27.6%), and stage IV—42 (21.9%).

Patients who had received neoadjuvant treatment before surgery were excluded from the study.

### 2.2 DNA extraction and quantitative PCR assay

DNA was extracted from formalin-fixed, paraffin-embedded (FFPE) blocks of primary colorectal tumors. Genomic DNA was isolated using the DNA-Tissue-M kit (TestGen, Moscow, Russia) according to the manufacturer’s protocol. Briefly, 10-µm FFPE sections were deparaffinized using sequential xylene–ethanol washes (ErgoProduction, Moscow, Russia), followed by tissue lysis in a proteinase K–containing buffer (TestGen, Moscow, Russia). After lysis, the lysate was incubated at 90 °C for 1 h to reverse formalin-induced cross-links. DNA was then captured on magnetic beads, washed, and eluted in TE buffer (TestGen, Moscow, Russia). DNA quality was assessed by spectrophotometry (A260/A280 ≥ 1.7). At the qPCR stage, a pre-specified assay-acceptance threshold of total bacterial load ≥10^5 copies per reaction was applied. This threshold was derived from the manufacturer’s workflow for the intended stool-based application of the assay, in which total bacterial mass not lower than 10^5 is used as an indicator of adequate sampling and DNA extraction; in the present FFPE study, it was used only as an operational assay-acceptance criterion. In the present cohort, all samples met this threshold, and no samples were excluded on this basis. We therefore report total bacterial load as an analyzed microbial variable rather than as an independent host-based internal control.

The Colonoflor Premium assay is a commercial qPCR kit originally intended for fecal samples and was applied here in an exploratory manner to FFPE tumor tissue. According to the manufacturer’s working instruction, the assay includes built-in positive and negative controls for each run, uses a total reaction volume of 35 μl including 5 μl of DNA template, and is interpreted with the aid of the manufacturer-provided quantitative analysis file. The user documentation provides the composition of the target panel and manufacturer-declared analytical characteristics, including analytical sensitivity in the range of 10^4–10^5 copies/ml depending on the target, as well as operational assay specificity based on expected amplification in the presence of target DNA and absence of exponential fluorescence growth in its absence. Primer oligonucleotide sequences are not disclosed in the available manufacturer documentation and were therefore not available for reporting in the present manuscript. DNA quality was assessed by spectrophotometry, and A260/A280 ≥ 1.7 was used as the pre-specified criterion for downstream processing. Run validity was assessed according to the manufacturer’s control logic, including evaluation of positive and negative control samples for each assay series.

The quantitative microbiota burden in DNA samples was assessed by quantitative PCR (qPCR) using the Colonoflor Premium kit (Alpha-Lab, Russia). The assay panel comprised the following targets (30 bacterial taxa and *Candida* spp. as a fungal target): *Lactobacillus* spp.*, Bifidobacterium* spp.*, Escherichia coli, Bacteroides* spp.*, Faecalibacterium prausnitzii, Bacteroides thetaiotaomicron, Akkermansia muciniphila, Enterococcus* spp.*, enteropathogenic Escherichia coli, Klebsiella pneumoniae, Klebsiella oxytoca, Candida* spp.*, Staphylococcus aureus, Clostridium difficile, Clostridium perfringens, Proteus vulgaris/Proteus mirabilis, Citrobacter* spp.*, Enterobacter* spp.*, Fusobacterium nucleatum, Parvimonas micra, Salmonella* spp.*, Shigella* spp.*, Blautia* spp.*, Acinetobacter* spp.*, Eubacterium rectale, Streptococcus* spp.*, Roseburia inulinivorans, Prevotella* spp.*, Methanobrevibacter smithii, Methanosphaera stadmanae, Ruminococcus* spp., as well as total bacterial load.

Detected microorganisms and total bacterial load were quantified as absolute values (genome copies per PCR reaction), which were subsequently log-transformed during statistical analyses.

### 2.3 Statistical analysis

All analyses were performed in Python v3.12 using the openpyxl, pandas, numpy, scipy, statsmodels, and scikit-learn libraries, figures were generated with matplotlib.

Descriptive statistics were reported as follows: for continuous variables, we calculated n, mean ± SD, median [IQR; 25th–75th percentiles], minimum–maximum, and the 5th/95th percentiles. Normality within groups was assessed using the Shapiro–Wilk test. As the data deviated from normality, non-parametric methods were applied: the Mann–Whitney U test for two-group comparisons and the Kruskal–Wallis test for comparisons of more than two groups, followed by Dunn’s *post hoc* test (Shapiro and Wilk, 1965; Mann and Whitney, 1947; Kruskal and Wallis, 1952; Dunn, 1964). Multiple-comparison adjustment was performed using the Benjamini–Hochberg (BH) procedure with calculation of the false discovery rate (FDR; q values) - BH/FDR-adjusted q value (Benjamini and Hochberg, 1995).

For categorical variables and microbiota composition analyses in presence/absence mode, quantitative values were binarized and proportions were compared using the χ² test; for low expected counts in 2×2 tables, Fisher’s exact test was used (Fisher, 1922). Effect sizes were reported as odds ratios (ORs) with 95% confidence intervals (CIs) for 2×2 tables, or as Cramér’s V for Rx2/RxC tables. For Rx2 tables, pairwise comparisons of proportions (two-proportion z tests) were additionally performed. BH/FDR adjustment was applied separately for global tests and *post hoc* comparisons.

Associations between quantitative taxon measures and ordinal clinicopathological outcomes (stage/grade) were evaluated using univariable ordinal logistic regression (proportional odds; ordered logit) (McCullagh, 1980). Outcomes were coded as ordered variables, taxon values were transformed as log10(1+X) and standardized (z-scored), with effects interpreted per 1 SD increase. Models were fitted in statsmodels (OrderedModel, distr=“logit”) using the BFGS optimizer (maxiter=400). We extracted β, SE, Wald z, and two-sided p values; ORs were calculated as exp(β), with 95% CIs computed as exp(β ± 1.96·SE). Multiple testing across taxa was controlled using BH/FDR. Were generated Forest plots and exploratory visualizations were generated for significant taxa.

For ordinal outcomes in qualitative (presence/absence) mode, univariable ordered logit models were fitted with the binary predictor Detect (McCullagh, 1980). Robust standard errors (HC1) were used by default. Reporting included β, ORs with 95% confident intervals (Cis), Wald z test p values, and BH/FDR-adjusted q values. Detection proportions across stage/grade levels were additionally visualized with 95% Cis (Wilson) (Wilson, 1927) and a smoothed logistic trend line.

The predictive value of taxon combinations for binary outcomes (early vs late stage, invasive vs non-invasive cancer, low vs high grade) was assessed using regularized logistic regression. For each taxon, two features were constructed: Detect (0/1) and Abundance = log10(1+count). Features were standardized (StandardScaler) within each fold: scaling parameters were estimated on the training split and applied to the corresponding test split. Signature selection used L1-penalized logistic regression (LASSO; sklearn LogisticRegression, solver=“saga”, class_weight=“balanced”) (Tibshirani, 1996), with the inverse regularization strength C tuned over a grid from 10^-4 to 10^4 via nested cross-validation (StratifiedKFold) (Cawley and Talbot, 2010), optimizing ROC-AUC while constraining signature size to ≤7 taxa (with each taxon represented by up to two features: Detect and Abundance). If no non-empty signature of ≤7 taxa was obtained within the grid, a fallback strategy was applied: the model with the best AUC was selected, the top 7 features were retained, and the model was refit with L2 regularization on the reduced feature set. Generalization performance was evaluated using external RepeatedStratifiedKFold (5 folds × 3 repeats). ROC-AUC, sensitivity, and specificity were computed on held-out folds. The classification threshold was chosen on the training split by maximizing Youden’s index. An aggregated confusion matrix and a summary of metrics with 95% empirical intervals (2.5th–97.5th percentiles of the fold-wise metric distributions) were reported. Feature stability was assessed by the frequency with which a given taxon was included in the selected signature. The final (“deployed”) model was trained on the full dataset, and the resulting signature (non-zero coefficients and effect direction) was recorded.

To compare within-panel profile distances across groups, an Aitchison approach was used: centered log-ratio (CLR) transformation with a pseudocount was applied and Euclidean distances were computed in CLR space (CLR-Euclidean) (Aitchison, 1982). As a sensitivity analysis, Bray–Curtis distances were calculated using within-panel proportions (normalization to the sum of panel taxa) (Bray and Curtis, 1957). Total bacterial load was not included in within-panel profile calculations. Ordination was performed using PCoA (Gower, 1966). Group differences were tested by PERMANOVA (pseudo-F, R², and permutation-based p values) (Anderson, 2001), and homogeneity of dispersions was assessed using PERMDISP (Anderson, 2006). Results were visualized as PCoA plots for both distance metrics.

As a sensitivity analysis for the grade-related findings, we repeated the principal two-group analyses after excluding G3 cases and restricting the comparison to G1 versus G2 tumors. This sensitivity analysis was performed only for the two pre-specified headline microbial features identified in the primary analysis: total bacterial load and *Ruminococcus* spp. detection. Because this was a focused sensitivity analysis of two pre-specified features rather than a genome- or panel-wide re-screening step, unadjusted p values are reported.

To assess whether the principal grade-associated microbial features were independent of major clinicopathological covariates, additional multivariable binary logistic regression models were fitted for the G2–G3 versus G1 comparison. These adjusted models were restricted to the two pre-specified headline features identified in the primary analysis: total bacterial load and Ruminococcus spp. detection. The covariates included age, sex, tumor location (colon vs rectum), and AJCC stage.

### 2.4 Ethical approval and informed consent statements

The study was conducted in accordance with the principles of the 1975 Helsinki Declaration and its 2013 version. The research protocol №8 was approved by the local ethical committee of the Petrovsky National Research Center of Surgery on May 23, 2025. All study participants provided written informed consent.

## 3 Results

A statistical analysis of the Colonoflor qPCR panel results was performed to identify patterns associated with each major clinicopathological grouping—tumor differentiation grade and disease stage. In addition, primary tumor T categories were analyzed separately, as only the primary tumor lesion was examined by qPCR.

Taxa with zero detection rates were excluded from further analyses. The final panel subjected to analysis comprised Total bacterial load and the following taxa: *Lactobacillus* spp.*, Bifidobacterium* spp.*, Escherichia coli, Bacteroides* spp.*, Faecalibacterium prausnitzii, Bacteroides thetaiotaomicron, Akkermansia muciniphila, Klebsiella pneumoniae, Staphylococcus aureus, Clostridium perfringens, Proteus vulgaris/Proteus mirabilis, Citrobacter* spp.*, Enterobacter* spp.*, Fusobacterium nucleatum, Blautia* spp.*, Acinetobacter* spp.*, Streptococcus* spp.*, Eubacterium rectale, Roseburia inulinivorans, Prevotella* spp.*, Methanobrevibacter smithii*, and *Ruminococcus* spp.

Detection frequencies varied across taxa. Both quantitative and qualitative (presence/absence) analyses were performed for all taxa, except for Total bacterial load, which was evaluated only quantitatively. Interpretation took detection frequency into account: for rare taxa, qualitative (presence/absence) analyses were considered more informative. Taxon detection frequencies are summarized in **Table 1**.

**Table 1:** Taxon detection frequencies in CRC tumor samples.

Taxa	Detection frequencies
Total bacterial load	1
Lactobacillus spp	0.1
Bifidobacterium spp	0.625
Escherichia coli	0.9125
Bacteroides spp	0.96875
Faecalibacterium prausnitzii	0.54375
Bacteroides thetaomicron	0.19375
Akkermansia muciniphila	0.0875
Klebsiella pneumoniae	0.04375
S aureus	0.08125
Clostridium perfringens	0.05625
Proteus vulgaris or mirabilis	0.025
Citrobacter spp	0.05625
Enterobacter spp	0.2
Fusobacterium nucleatum	0.25625
Blautia spp	0.1
Acinetobacter spp	0.94375
Streptococcus spp	0.11875
Eubacterium rectale	0.05
Roseburia inulinivorans	0.24375
Prevotella spp	0.2375
Methanobrevibacter smithii	0.0625
Ruminococcus spp	0.13125

All 192 samples met the pre-specified assay-acceptance threshold (total bacterial load ≥10^5 copies per reaction); log10 total bacterial load ranged from 8.5 to 11.6. No exclusions occurred either overall or within the investigated subgroups. Because all samples met the pre-specified assay-acceptance threshold, this threshold was non-binding in the present cohort and did not alter the analytic sample.

### 3.1 Analysis of groups with different degrees of differentiation (grade) of primary epithelial malignancies.

For the analysis of tumor differentiation (grade), two groups were defined: well-differentiated colorectal adenocarcinomas—Low grade (G1) (N = 108) and moderately/poorly differentiated colorectal adenocarcinomas—High grade (G2–G3) (N = 84).

**Figure 1:**
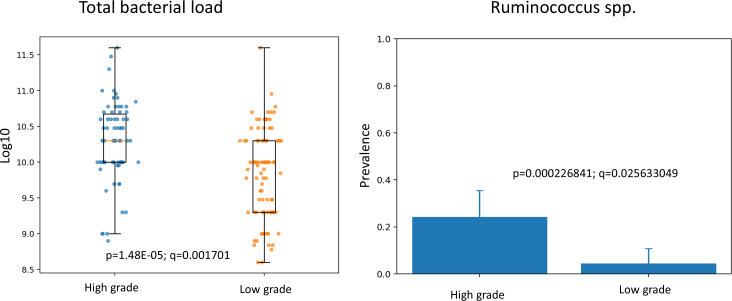
Comparative plot of total bacterial load (left) and the detection frequency of *Ruminococcus* spp. (right) across different grades of colon and rectal adenocarcinomas.

In the quantitative taxon analysis, Total bacterial load was higher in high-grade tumors than in low-grade tumors (median log10 10.3 [10.00–10.68] vs 10.0 [9.30–10.30], respectively); p (Mann–Whitney U)=1.47934×10^-5, q (Benjamini–Hochberg FDR)=0.0017. In addition, high-grade tumors showed a higher level of *Ruminococcus* spp. than low-grade tumors, most clearly demonstrated in the qualitative (presence/absence) analysis: the detection frequency of *Ruminococcus* spp. was 0.24 (95% CI 0.16–0.35) versus 0.04 (95% CI 0.017–0.109), respectively; p (χ² test, 2×2)=0.000227, q=0.0256 (**Figure 1**).

A trend toward higher levels of *Escherichia coli* and *Bacteroides* spp. was observed in the G2–G3 group; however, these associations did not remain significant after correction for multiple testing (FDR): p=0.0047, q=0.1224 and p=0.00275, q=0.1054, respectively.

In the qualitative analysis of taxa with low detection frequencies, we observed a trend toward increased detection of *Clostridium perfringens*, *Fusobacterium nucleatum*, *Blautia* spp., and *Roseburia* detected in the G2–G3 group; however, these associations did not withstand multiple-testing correction: p=0.01076, q=0.4054; p=0.0269, q=0.6078; p=0.0336, q=0.6327; and p=0.0100, q=0.4054, respectively.

**Figure 2:**
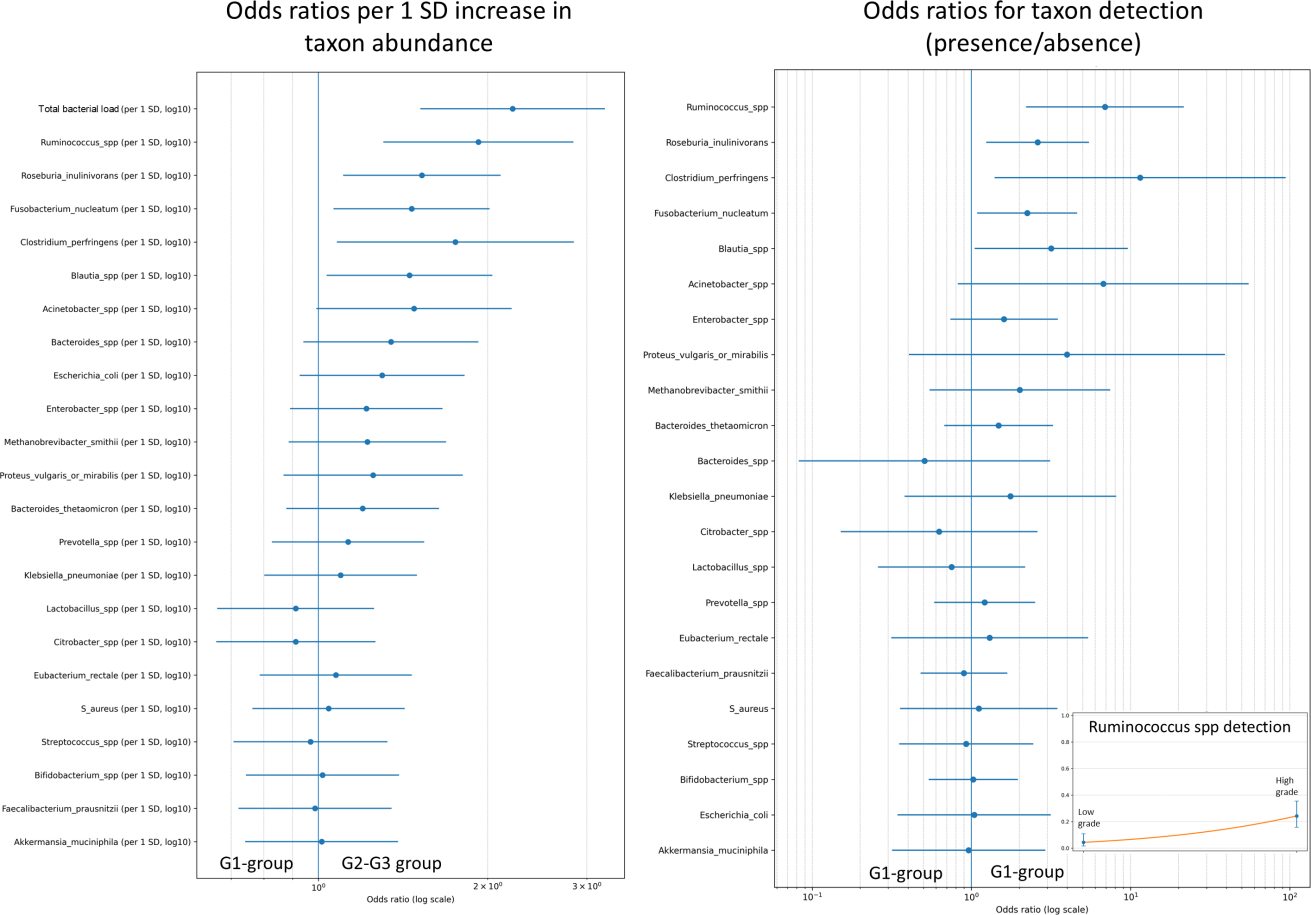
Forest plot of regression analyses assessing the association between taxon abundance (left) and taxon detection (presence/absence; right) and adenocarcinoma grade. For each taxon, the odds ratio (OR) with the corresponding 95% confidence interval is shown. For *Ruminococcus* spp., an additional plot illustrates the change in detection frequency between the G1 and G2–G3 groups (lower left corner).

Regression analyses of taxon abundance and detection corroborated the comparative statistics. Specifically, higher Total bacterial load was associated with the G2–G3 group malignant tumors (OR = 2.2146 (95% CI 1.5173–3.2324); p=3.76524×10^-5; q=0.000866005), and increased abundance and detection of *Ruminococcus* spp. were also associated with the G2–G3 group relative to G1 (for detection: OR = 6.896 (95% CI 2.2017–21.5998); p=0.000916; q=0.0201). A similar trend toward higher abundance and detection in the G2–G3 group was noted for several other taxa—*Roseburia inulinivorans*, *Clostridium perfringens*, *Fusobacterium nucleatum*, and *Blautia* spp.—for which p<0.05, although none remained significant after FDR adjustment (all q>0.1) (**Figure 2**).

To assess whether the grade-associated findings were driven mainly by the small G3 subgroup, we performed a sensitivity analysis restricted to G1 versus G2 tumors. In this restricted comparison, the association for total bacterial load remained significant (OR = 2.389, 95% CI 1.5894 – 3.5916, p =2.81×10^-5). The association for Ruminococcus spp. detection also remained significant (OR = 6.862, 95% CI 2.15-21.86, p = 0.001124). These results indicate that the principal grade-related signals were preserved after exclusion of G3 cases and were therefore not solely attributable to the small poorly differentiated subgroup.

To further assess whether the principal grade-associated findings were independent of basic clinicopathological covariates, we fitted multivariable binary logistic regression models for the G2–G3 versus G1 comparison, adjusting for age, sex, tumor location, and AJCC stage. In these adjusted models, total bacterial load remained significantly associated with the G2–G3 group (OR = 2.68, 95% CI 1.49–4.81, p = 0.00098). Ruminococcus spp. detection also remained significantly associated with the G2–G3 group after adjustment (OR = 10.23, 95% CI 1.08–96.75, p = 0.0425). Thus, the two principal grade-related microbial signals persisted after covariate adjustment.

A predictive analysis of taxon combinations was performed. To classify tumors as G2–G3 versus G1, a LASSO-penalized logistic regression model with nested cross-validation was fitted. For predicting high-grade CRC, the resulting regularized logistic model demonstrated moderate discriminative performance under external evaluation within nested stratified cross-validation (Repeated Stratified K Fold 5×3). The mean ROC-AUC was 0.707 (SD 0.095; empirical 95% interval 0.565–0.832). Using a classification threshold selected on the training split of each outer run by maximizing Youden’s index, the mean sensitivity was 0.529 (95% interval 0.214–0.832) and the mean specificity was 0.670 (95% interval 0.389–0.925).

**Figure 3:**
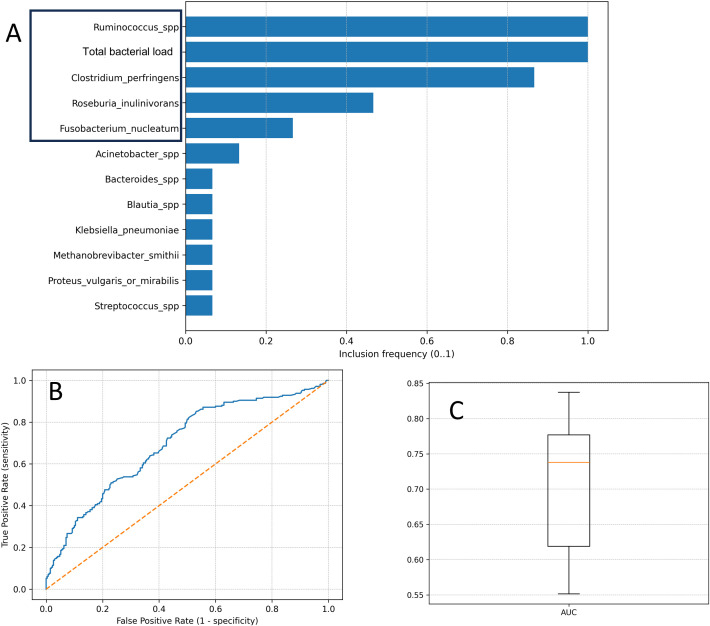
Predictive performance of taxon-combination models. **(A)** Feature-selection stability plot showing taxon inclusion frequencies; the four taxa retained in the final signature are highlighted. **(B)** ROC curve of the derived predictive model. **(C)** Distribution of ROC-AUC across cross-validation runs, shown as the median AUC (center line), interquartile range (box), and empirical 95% interval (whiskers).

The final “deployed” signature comprised four predictors: Total bacterial load as a quantitative feature and detection (presence) of *Ruminococcus* spp., *Clostridium perfringens*, and *Roseburia inulinivorans* as qualitative features. All predictor coefficients were positive, consistent with an increased probability of meeting the high-grade threshold with increasing abundance and/or presence of the corresponding features. Feature-selection stability, assessed by the frequency of inclusion in the signature across outer runs, showed high stability for Total bacterial load and *Ruminococcus* spp. (both 15/15), and for *Clostridium perfringens* (13/15), whereas *Roseburia inulinivorans* was included less consistently (7/15). Notably, for rare features—particularly detection of C. perfringens (detection rate ~5.6%)—selection stability may be less reliable; therefore, the contribution of rare taxa should be interpreted as hypothesis-generating and requires independent validation (**Figure 3**).

**Figure 4:**
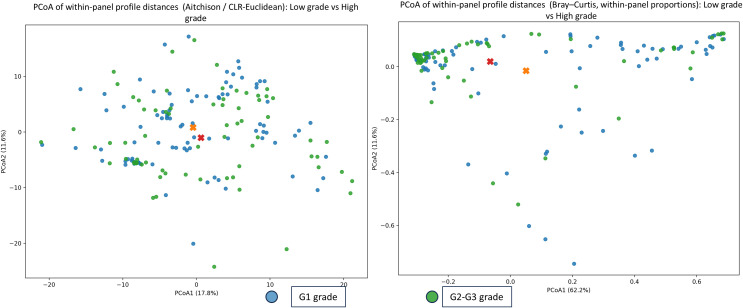
Within-panel profile-distance metrics of microbiological profiles in tumors stratified by differentiation grade. Aitchison (CLR-Euclidean) distances are shown on the left, with no evident visual clustering between groups (PERMANOVA: p=0.094; R²=0.009). Bray–Curtis distances are shown on the right and likewise demonstrate no pronounced group separation (PERMANOVA: p=0.030; R²=0.022).

When comparing within-panel profile distances between the G2–G3 group and G1, no clear separation of group centroids was observed for the primary Aitchison metric (CLR-Euclidean) (PERMANOVA: p=0.094; R²=0.009), whereas Bray–Curtis distances showed a nominally significant difference with a small effect size (PERMANOVA: p=0.030; R²=0.022). PERMDISP indicated statistically significant differences in within-group dispersion for both metrics (Aitchison: p=0.010; Bray–Curtis: p=0.006), suggesting differences in profile heterogeneity between groups and warranting cautious interpretation of the PERMANOVA results. Visually, no pronounced group separation was evident on the PCoA plots (**Figure 4**).

### 3.2 Analysis of groups stratified by primary tumor stage

Because the study was conducted on primary tumor tissue, one of the objectives was to assess whether the detected taxa were associated with the primary tumor stage (T category according to the TNM classification). The cohort included T1 (n=3), T2 (n=32), T3 (n=116), and T4 (n=41) cases. In addition, two aggregated groups were defined: “non-invasive cancer”—tumors without invasion into the muscular layer of the bowel wall (T1–T2; n=35) and “invasive cancer”—tumors with invasion into the muscular layer (T3–T4; n=157). Ordinal logistic regression analyses were performed without the T1 category because this group comprised only three cases.

**Figure 5:**
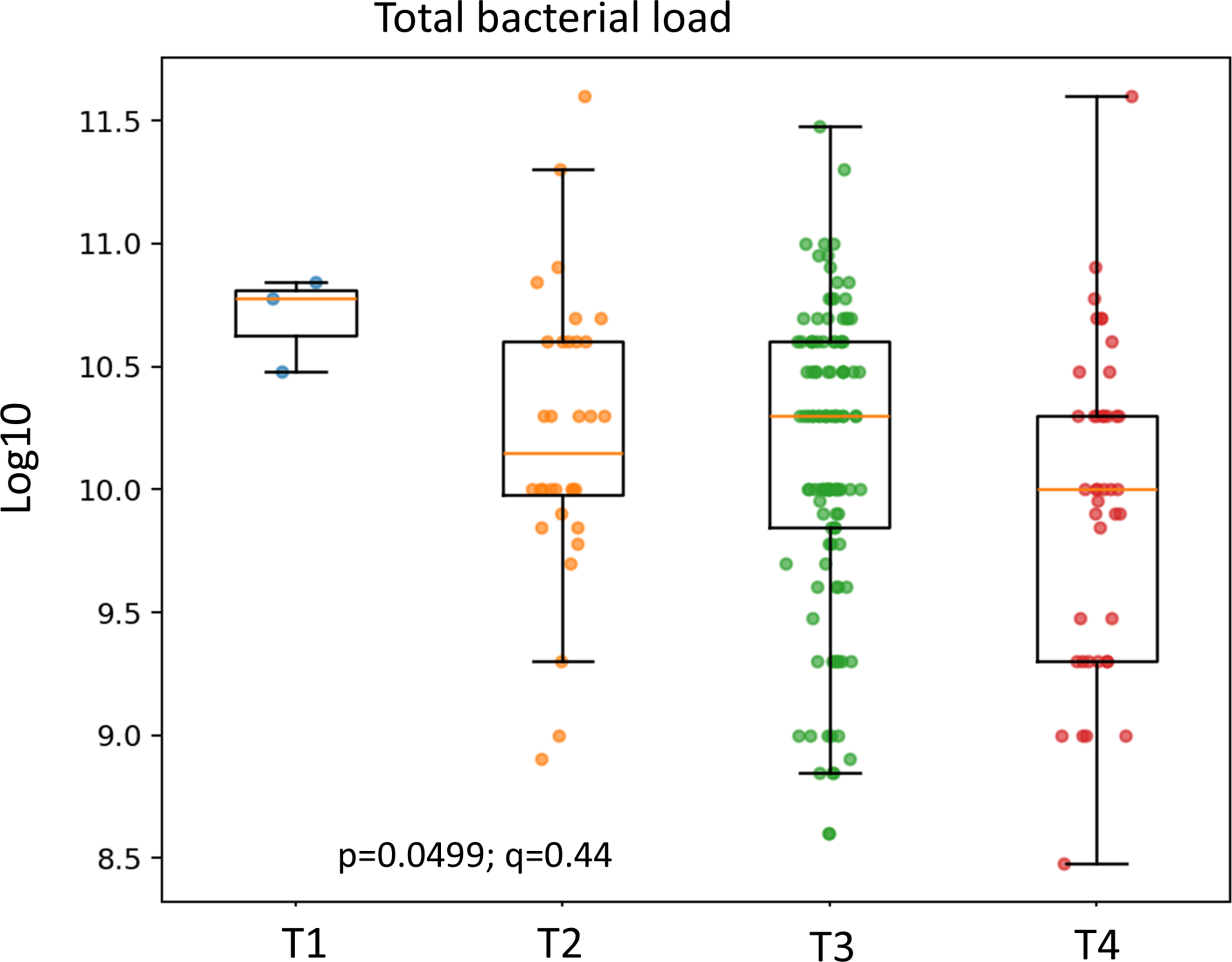
Comparative plot of total bacterial load across primary tumor T categories. Although the overall between-group difference reached nominal significance, no statistically significant differences were observed after adjustment for multiple comparisons and subsequent pairwise *post hoc* testing.

Total bacterial load showed a trend toward decreasing values with increasing tumor stage (T1 to T4), with median log10 values of 10.78 [10.65–10.81], 10.17 [9.98–10.60], 10.30 [9.85–10.60], and 10.00 [9.30–10.30], respectively. The global comparison was nominally significant (Kruskal–Wallis p=0.0499); however, after multiple-testing correction the association was not significant (q=0.44), and *post hoc* pairwise comparisons did not reveal even nominally significant differences between stages (**Figure 5**).

Comparisons of taxa in the qualitative (presence/absence) mode did not reveal statistically significant differences between T1–T4. Likewise, comparison between the non-invasive (T1–T2) and invasive (T3–T4) groups showed no significant between-group differences.

**Figure 6:**
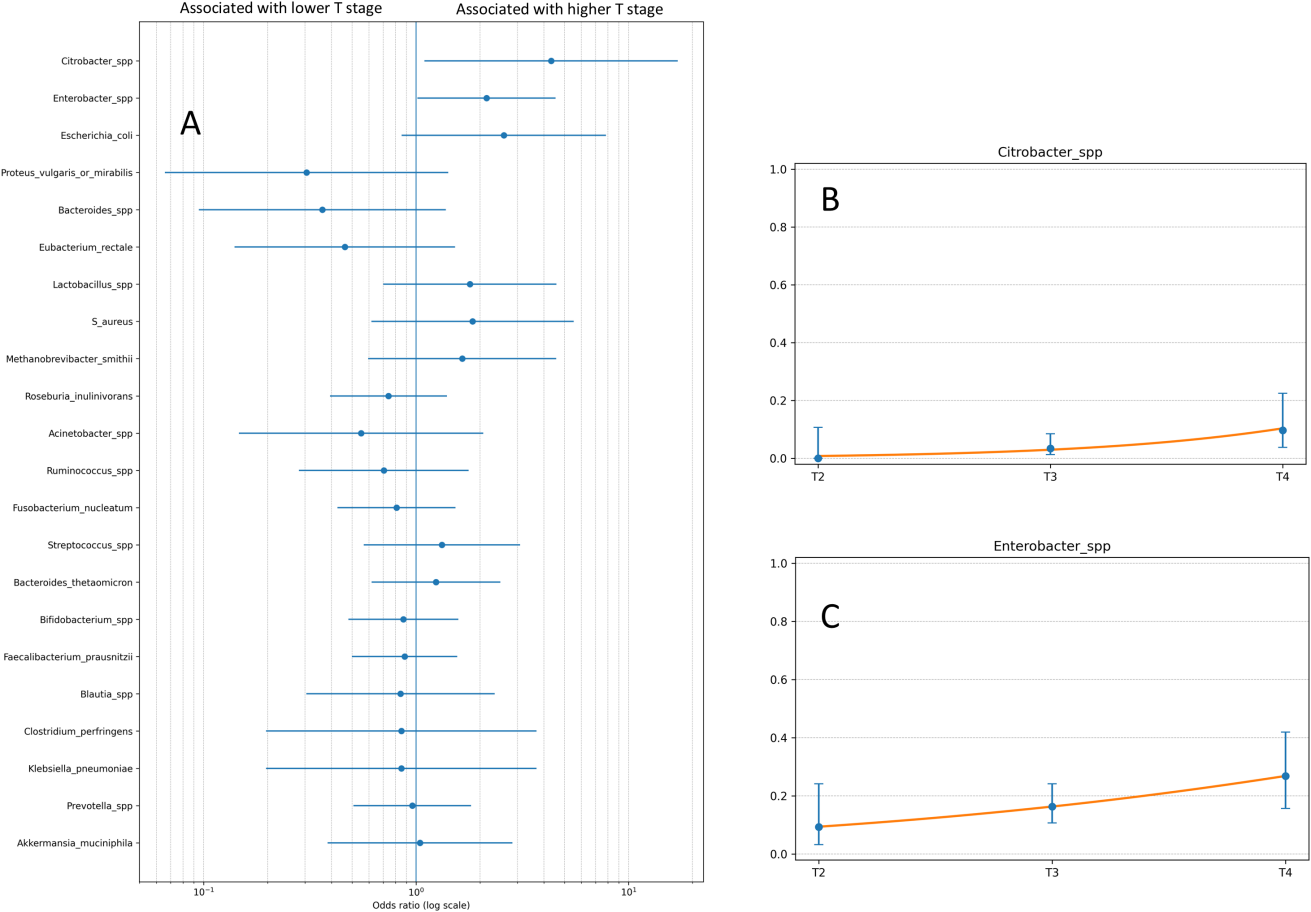
Ordinal regression results for taxon detection as a function of primary tumor T stage. **(A)** Forest plot of the ordered logistic regression (qualitative, presence/absence) evaluating associations between taxon detection and T stage. The odds ratios (ORs) and 95% confidence intervals for *Citrobacter* spp. and *Enterobacter* spp. were nominally consistent with higher detection frequencies at more advanced T categories. **(B)** Detection frequency of *Citrobacter* spp. across T stages. **(C)** Detection frequency of *Enterobacter* spp. across T stages.

Ordinal logistic regression identified a trend toward a positive association between T stage and both abundance and detection of *Citrobacter* spp. and *Enterobacter* spp. The overall detection frequencies of *Citrobacter* spp. and *Enterobacter* spp. were 5.6% and 20%, respectively (**Table 1**); therefore, these results are more appropriately interpreted in qualitative terms (detection frequency). Accordingly, the odds ratio (OR) for *Citrobacter* spp. was 4.32 (95% CI 1.095–17.039; p=0.0366; q=0.504), and for *Enterobacter* spp. was 2.145 (95% CI 1.014–4.539; p=0.046; q=0.504). As shown, these trends did not remain significant after adjustment for multiple testing (BH/FDR q values) (**Figure 6**).

A predictive analysis of taxon combinations was performed to classify patients into non-invasive (T1–T2) and invasive (T3–T4) cancer groups using a regularized logistic regression model (L1/LASSO). The final signature comprised seven taxa, including Total bacterial load (abundance), *Escherichia coli* (detection + abundance), *Proteus vulgaris Proteus mirabilis* (detection + abundance), *Citrobacter* spp. (detection + abundance), *Enterobacter* spp. (detection + abundance), *Acinetobacter* spp. (detection + abundance), and *Methanobrevibacter smithii* (detection + abundance). Coefficients in the final model showed mixed directions: several predictors had positive effects (e.g., the abundance component of *E*. *coli*, both detection and abundance components of *M*. *smithii*, as well as *Citrobacter* spp. and *Enterobacter* spp.), whereas others had negative effects (for Total bacterial load abundance, *Proteus vulgaris Proteus mirabilis*, and detection of *E*. *coli* and *Acinetobacter* spp.). For some taxa, the sign of the effect differed between qualitative (detection) and quantitative (abundance) representations.

**Figure 7:**
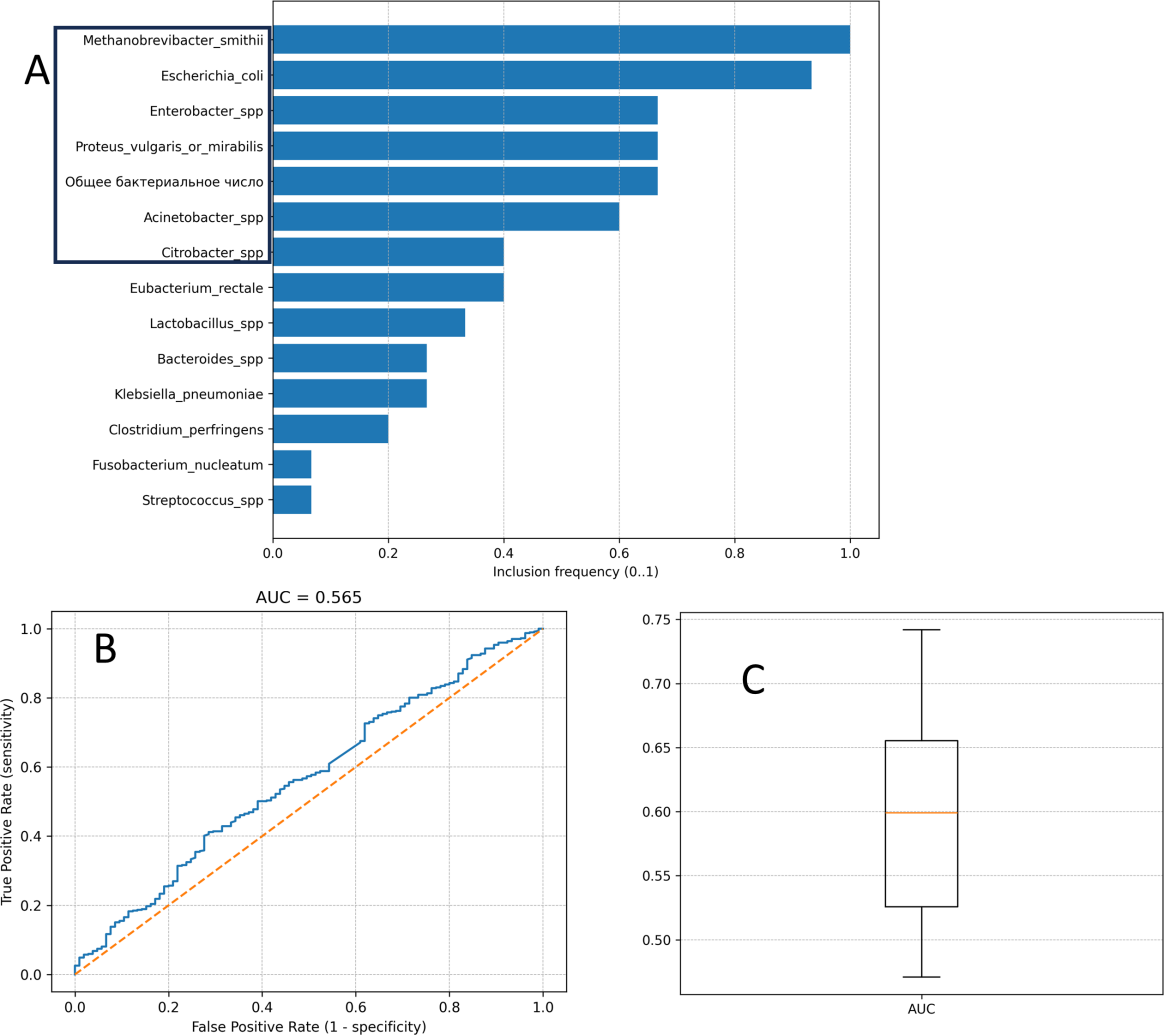
Predictive performance of the taxon-combination model for distinguishing non-invasive (T1–T2) from invasive (T3–T4) primary tumors. **(A)** Feature-selection stability plot showing taxon inclusion frequencies; the seven taxa retained in the final signature are highlighted. **(B)** ROC curve of the derived predictive model. **(C)** Distribution of ROC-AUC across cross-validation runs, shown as the median AUC (center line), interquartile range (box), and empirical 95% interval (whiskers).

The model achieved a mean ROC-AUC of 0.593 (95% CI 0.475–0.720; SD 0.080), with mean sensitivity of 0.560 and specificity of 0.533. Overall, the derived model demonstrated low discriminative performance for the T1–T2 versus T3–T4 comparison in the present dataset. The results nonetheless recapitulated trends observed previously, including a positive association of invasive T categories with detection of Citrobacter spp. and Enterobacter spp., and a negative association with Total bacterial load abundance (**Figure 7**).

**Figure 8:**
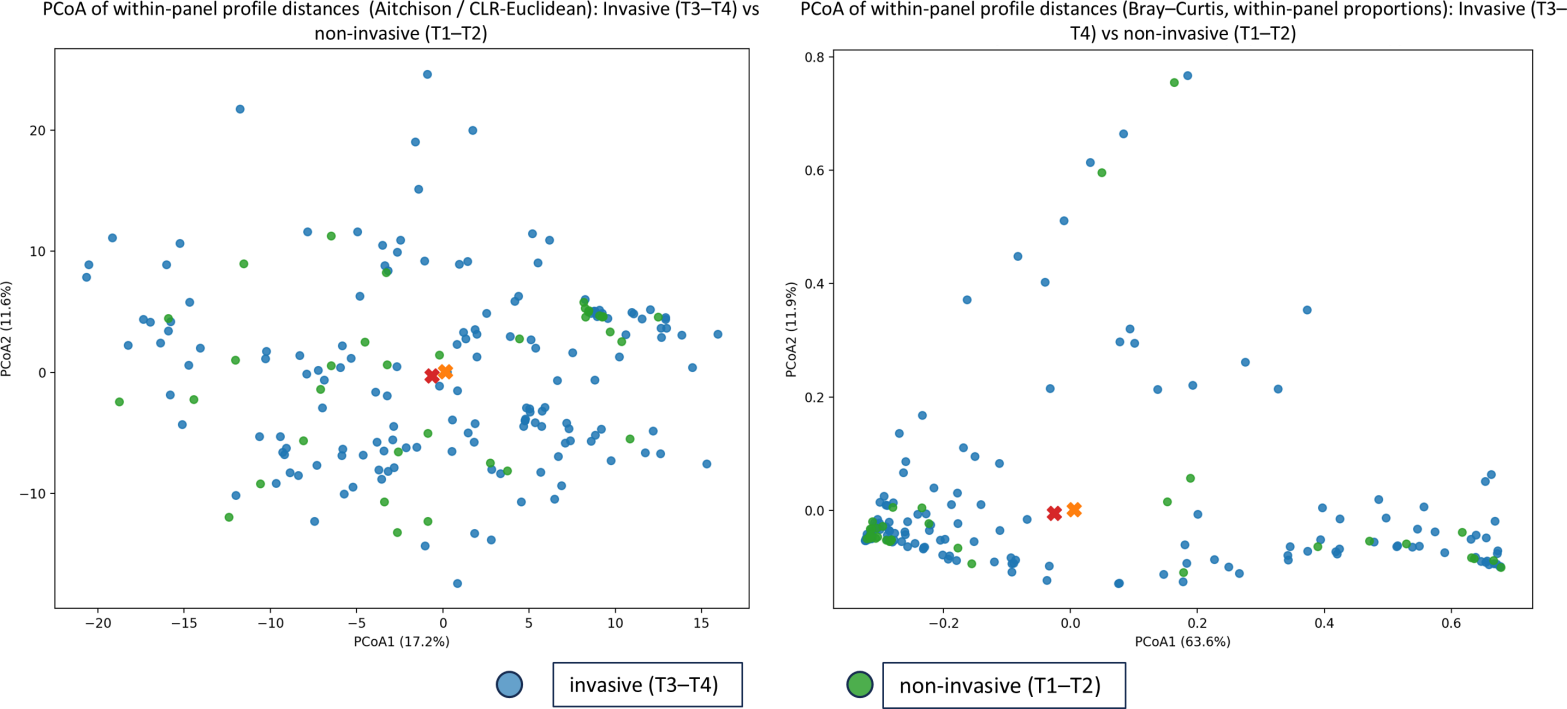
Within-panel profile-distance metrics of microbiological profiles in tumors stratified by invasiveness (non-invasive, T1–T2 vs invasive, T3–T4). Aitchison (CLR-Euclidean) distances are shown on the left, with no evident visual clustering between groups (PERMANOVA: p=0.64; R²=0.004). Bray–Curtis distances are shown on the right and likewise demonstrate no pronounced group separation (PERMANOVA: p=0.82; R²=0.002).

To determine whether distinct microbiome patterns characterize non-invasive versus invasive cancer, analysis of within-panel profile distances was performed. No significant differences in panel-derived microbial profiles were observed: PERMANOVA for Aitchison (CLR-Euclidean) distances (F = 0.816, R²=0.004, p=0.6400) and for Bray–Curtis distances (F = 0.307, R²=0.002, p=0.8220). PERMDISP likewise showed no evidence of dispersion differences between groups (Aitchison p=0.7100; Bray–Curtis p=0.7970). Consistently, no visual separation of groups was apparent on the PCoA plots, and no global microbial-profile pattern discriminating non-invasive from invasive CRC was identified (**Figure 8**).

### 3.3 Analysis of groups stratified by colorectal cancer stage

To evaluate associations between the detected taxa and disease stage, patients were stratified into four groups according to the American Joint Committee on Cancer (AJCC) TNM Staging System (8th ed., 2017 for colon cancer; 8th ed., 2017 for rectal cancer) (**Table 2**).

**Table 2:** American Joint Committee on Cancer (AJCC) TNM staging system for colon cancer (8th ed., 2017) and rectal cancer (8th ed., 2017).

Stage	T	N	M
Stage 0	Tis	N0	M0
Stage I	T1, T2	N0	M0
Stage IIA	T3	N0	M0
Stage IIB	T4a	N0	M0
Stage IIC	T4b	N0	M0
Stage IIIA	T1–T2	N1/N1c	M0
Stage IIIA	T1	N2a	M0
Stage IIIB	T3–T4a	N1/N1c	M0
Stage IIIB	T2–T3	N2a	M0
Stage IIIB	T1–T2	N2b	M0
Stage IIIC	T4a	N2a	M0
Stage IIIC	T3–T4a	N2b	M0
Stage IIIC	T4b	N1–N2	M0
Stage IVA	Any T	Any N	M1a
Stage IVB	Any T	Any N	M1b
Stage IVC	Any T	Any N	M1c

Stage I (including Stage I), Stage II (including Stage IIA, Stage IIB, and Stage IIC), Stage III (including Stage IIIA, Stage IIIB, and Stage IIIC), and Stage IV (including Stage IVA, Stage IVB, and Stage IVC).

The study included 25 cases with stage I disease, 72 with stage II, 53 with stage III, and 42 with stage IV. In addition, two aggregated groups were formed: an “early cancer” group comprising stages I–II (n=97) and a “late cancer” group comprising stages III–IV (n=95).

Quantitative analyses indicated that Total bacterial load differed across disease stages (Kruskal–Wallis test p=0.0064); however, this difference did not remain significant after adjustment for multiple testing (q=0.122). Pairwise comparisons identified differences only between stage IV and the other stages, suggesting a mild trend toward lower Total bacterial load at stage IV; this trend was likewise not supported after multiple-testing correction. Median Total bacterial load log10 values were: stage I 10.3 (10.0–10.7), stage II 10.0 (9.95–10.6), stage III 10.3 (9.9–10.6), and stage IV 9.9 (9.3–10.3). As shown, the trend is weak and not fully consistent between stages I and III. A similar pattern—lower values at the final stage—was observed for quantitative Escherichia coli abundance, with a nominally significant Kruskal–Wallis test that did not persist after BH/FDR correction (p=0.019; q=0.252). Median values were: Stage I 5.0 (4.6–6.0), Stage II 5.3 (4.83–6.0), Stage III 5.3 (4.85–6.3), and Stage IV 4.81 (4.48–5.3). Pairwise comparisons suggested a tendency toward lower E. coli abundance at stage IV compared with stages II and III, again without confirmation after BH/FDR multiple-testing correction.

**Figure 9:**
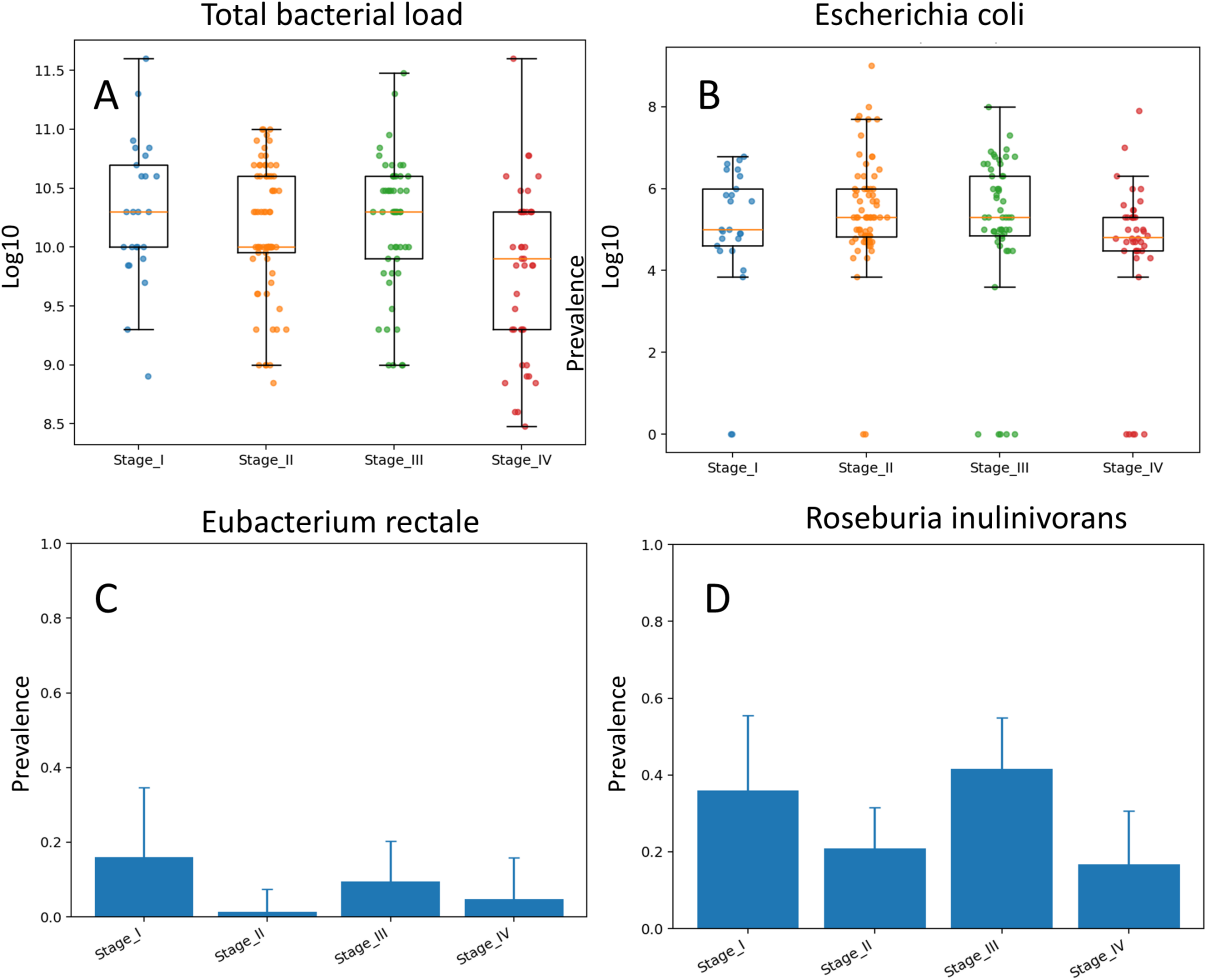
Comparative plots of total bacterial load **(A)**, *Escherichia coli* abundance **(B)**, and detection frequencies of Eubacterium rectale and *Roseburia inulinivorans* **(C, D)**, respectively across colorectal cancer stages from stage I to stage IV.

In qualitative analyses, χ² testing across stages (Rx2 contingency tables) suggested between-stage differences for *Eubacterium rectale* and *Roseburia inulinivorans*, but neither association withstood BH/FDR correction (p=0.045, q=0.731; and p=0.017, q=0.484, respectively). Beyond the limited statistical support, the observed between-stage differences were difficult to interpret due to non-monotonic changes in detection proportions across the ordered stage categories (**Figure 9**).

Comparison of the early-stage (stages I–II) and late-stage (stages III–IV) groups did not reveal statistically significant differences.

Ordinal logistic regression indicated a trend toward an inverse association between disease stage and Total bacterial load (OR < 1; OR = 0.659 [95% CI 0.503–0.864]; p=0.0025). However, this trend was not confirmed after multiple-testing correction (FDR), although it approached the 0.05 threshold (q=0.058).

**Figure 10:**
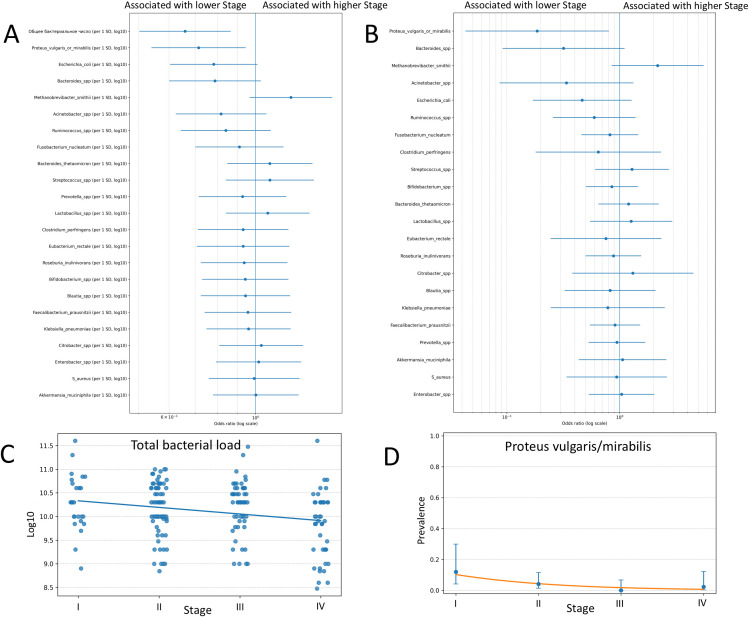
Ordinal regression results for taxa as a function of colorectal cancer stage. **(A)** Forest plot of the ordered logistic regression evaluating associations between taxon abundance (quantitative analysis) and disease stage. **(B)** Forest plot of the ordered logistic regression evaluating associations between taxon detection (qualitative, presence/absence) and disease stage. **(C)** Plot showing the trend in total bacterial load across disease stages. **(D)** Detection frequency of *Proteus vulgaris*/*Proteus mirabilis* across disease stages.

*Proteus vulgaris/Proteus mirabilis* was interpreted in the qualitative (presence/absence) mode given its low overall detection frequency (mean detection rate 2.5%; **Table 1**). An inverse trend between *Proteus vulgaris/Proteus mirabilis* detection and disease stage was observed (OR = 0.186 [95% CI 0.043–0.802]; p=0.0241), but this association did not remain significant after FDR correction (q=0.53) (**Figure 10**).

Logistic regression comparing early- versus late-stage disease did not identify any nominally significant associations.

The selection of a taxon-combination model to classify patients with late-stage disease using regularized logistic regression yielded a final signature comprising four predictors: Total bacterial load (abundance), *Escherichia coli* (abundance), *Proteus vulgaris/Proteus mirabilis* (detection), and *Methanobrevibacter smithii* (abundance). Coefficients were negative for the first three predictors and positive for *M. smithii*. Across external test runs, the mean ROC-AUC was 0.457 (95% CI 0.346–0.546; SD 0.065), with mean sensitivity of 0.439 and specificity of 0.489. Predictor inclusion frequencies across outer runs were 0.667 for *Proteus vulgaris/Proteus mirabilis* and Total bacterial load, and 0.533 for *E. coli* and *M. smithii*. These findings indicate low exploratory classification performance, suggesting that this targeted qPCR panel has limited ability to distinguish stage I–II from stage III–IV disease in the present dataset.

Analysis of within-panel profile distances led to the same conclusion. No significant differences in panel-derived profile distances were observed: PERMANOVA was non-significant for both Aitchison (CLR-Euclidean) distances (F = 0.654, R²=0.003, p=0.829) and Bray–Curtis distances (F = 0.644, R²=0.003, p=0.492). PERMDISP likewise showed no statistically significant differences in dispersion (Aitchison p=0.166; Bray–Curtis p=0.100). Collectively, these results indicate that, within the selected panel, no robust panel-profile pattern discriminating early from late disease stages was detected.

## 4 Discussion

The present findings indicate that, within the investigated microorganism qPCR exploratory panel applied to primary tumor tissue, the most pronounced and statistically robust differences were associated not with disease stage or primary tumor T category, but with tumor differentiation grade. In particular, tumors in the G2–G3 group showed increased Total bacterial load and a higher detection frequency of *Ruminococcus* spp., with both associations remaining significant after correction for multiple comparisons. Importantly, these associations persisted in a sensitivity analysis restricted to G1 versus G2 tumors, indicating that the observed signal was not driven solely by the small G3 subgroup. Also, both associations remained statistically significant in multivariable models adjusted for age, sex, tumor location, and stage, although the confidence interval for Ruminococcus spp. detection was wide and should therefore be interpreted cautiously.

Tumor differentiation grade is an integrative and relatively broad characteristic that reflects a wide range of biological features, including properties of the local tumor microenvironment (e.g., increased inflammatory infiltrate, impaired barrier function, areas of necrosis/ulceration, and alterations of the mucous layer and tissue trophism). These features may plausibly facilitate bacterial colonization and/or the accumulation of bacterial DNA within tumor material.

In this context, total bacterial load may be interpreted not simply as a technical qPCR-derived quantity, but as a broad indicator of the degree of bacterial DNA accumulation within the tumor microenvironment. Several, not mutually exclusive, biological mechanisms may contribute to such a signal. First, less differentiated tumors may exhibit more pronounced barrier disruption, surface ulceration, necrotic change, and mucus-layer disorganization, thereby facilitating bacterial access to tumor tissue. Second, increased total bacterial load may reflect altered local conditions within the tumor, including impaired clearance of microbial material and greater exposure of tissue to luminal microorganisms. Third, it may also be indirectly linked to inflammatory and immune-cell infiltration, insofar as microbial products and bacterial DNA can accumulate within areas of active host–microbe interaction. At the same time, the present design does not allow discrimination between viable intratumoral colonization, transient bacterial translocation, and accumulation of extracellular or cell-associated bacterial DNA. Therefore, total bacterial load should be interpreted as an integrative tissue-associated microbial signal rather than as a direct measure of live bacterial biomass.

It should be noted that, in the literature, associations between the microbiota and CRC grade (degree of differentiation) are reported far less frequently than associations with stage (TNM) or tumor location. This is particularly true for studies using tumor tissue as the biospecimen, consequently, for most taxa the evidence base remains fragmented and is often limited by small subsets of poorly differentiated tumors.

In tumor-tissue studies, Fusobacterium nucleatum is most commonly discussed as a taxon associated with poorly differentiated tumors (Huangfu et al., 2021), however, these data are not unequivocal and differ across studies (Kulmambetova et al., 2025). Consistently, our results indicate that *F*. *nucleatum* shows only nominal trends toward an association with tumor grade.

The literature also reports other taxa associated with tumor grade (e.g., in poorly differentiated CRC: *Bifidobacterium*, “*norank*_*f:Oscillospiraceae*”, *Eisenbergiella*; in moderately differentiated CRC: *Megamonas*, *Erysipelotrichaceae*_*UCG-003*, and the genus *Actinomyces*). However, these findings were derived from metagenomic studies of intestinal contents rather than tumor tissue (Qi et al., 2022). Accordingly, these observations should be interpreted as hypothesis-generating and sensitive to statistical power and data structure—an important caveat that also applies to our study.

The panel-derived profile differences between grade groups, assessed by PCoA and distance-based analyses, did not show visible clustering (a small nominal effect was observed only for Bray–Curtis). Importantly, PERMDISP detected differences in within-group dispersion for both distance metrics, indicating differences in profile heterogeneity. This may suggest that tumors in the G2–G3 group exhibit greater variability in tissue-associated microbial profiles, consistent with the broader biological heterogeneity associated with higher-grade colorectal carcinomas (Karamchandani et al., 2025).

Model-based analyses corroborate this pattern: for the G2–G3 group, the regularized logistic regression model showed moderate discriminative performance (AUC ~0.71) and identified a taxon combination comprising Total bacterial load together with qualitative detection of *Ruminococcus* spp., *C*. *perfringens*, and *R*. *inulinivorans*. As an exploratory classification signature, this combination may have limited practical utility because of its relatively low sensitivity and specificity; however, it may support the relevance of the identified taxa.

In contrast to the differentiation-grade–focused analyses, attempts to build models for overall disease stage and primary tumor T category yielded low-performing results across multiple complementary statistical approaches. The nominal associations observed for invasion depth (T category) and/or disease stage—linked to Total bacterial load and several rare taxa such as *Citrobacter* spp., *Enterobacter* spp., and *Proteus vulgaris/Proteus mirabilis*—should be regarded as hypothesis-generating and require further confirmation.

Overall, considering the taxa that showed nominal associations with clinicopathological characteristics of CRC, Total bacterial load, interpreted as tissue bacterial burden, may be viewed as an integrative indicator of microbial colonization of the tissue microenvironment. However, at this level of analysis it is difficult to delineate specific links to distinct mechanisms of carcinogenesis (Shi et al., 2025).

The genus *Ruminococcus* spp., when considered in the context of CRC, is highly heterogeneous, and its role depends on the specific species involved (Shi et al., 2025), nevertheless, qPCR-based assessment of *Ruminococcus* spp. has been used in a previous study (Shakhpazyan et al., 2025), where a trend toward detection of this taxon in metastatic CRC was reported.

*Citrobacter* spp. and *Enterobacter* spp., which in our study showed nominal associations with more advanced primary tumors (T category), have been reported to be more frequently detected in tumor tissue and, in some studies, higher levels have been linked to poorer prognosis (Kushwaha et al., 2025; Shi et al., 2025), moreover, *Citrobacter rodentium* infection in mouse models enhances epithelial proliferation and promotes carcinogenesis in genetically predisposed animals (ApcMin/+) (Newman et al., 2001).

In biopsies from patients with adenomas and carcinomas, the presence of intracellularly localized *Proteus mirabilis* and *Proteus vulgaris* has been described (Wachsmannova et al., 2018), and the genus *Proteus* is generally considered to contribute to CRC pathogenesis (Liu et al., 2023a). In our study, *Proteus* was less frequently detected in tissue at later stages of colorectal adenocarcinoma; however, it was a rarely detected taxon overall (detection frequency 2.5%). In addition, we did not identify studies directly comparing *Proteus* detection in tumor tissue across stages. Therefore, the relationship and role of intratissue *P. mirabilis* and *P. vulgaris* in colorectal carcinogenesis remain hypothesis-generating.

Based on our data, it is evident that further progress in this research direction will require expanding the microorganism panel to obtain a more comprehensive view of the tumor-associated microbiome and to enable assessment of α-diversity metrics. Given the nature of the material examined in our study (histological tumor tissue specimens FFPE), 16S rRNA gene sequencing represents a promising approach, as it can provide a more complete characterization of the tumor-associated bacterial community required for evaluating integrative biodiversity measures (Schulz et al., 2025).

### 4.1 Study limitations

This study has several limitations. First, it was a retrospective study based on archived FFPE primary tumor material. In addition, the poorly differentiated subgroup (G3) was small (n = 10), which limits statistical power for analyses specifically centered on this category and reduces the precision of subgroup-specific inference. Although the principal grade-related signals remained significant in a sensitivity analysis restricted to G1 versus G2 tumors, the small size of the G3 subgroup remains an important limitation when interpreting findings across the full three-tier differentiation spectrum. Furthermore, the use of FFPE material introduces matrix-specific biases, including DNA fragmentation and formalin-related damage, which may reduce microbial DNA detectability and affect quantitative estimates for some targets. Second, no dedicated contamination-monitoring controls, such as extraction blanks or paraffin-only controls, were incorporated into the original workflow. Therefore, reagent- or environment-derived background signal (contamination) cannot be fully excluded. This limitation is particularly relevant for highly prevalent taxa in the present dataset, which should therefore be interpreted cautiously in the absence of dedicated contamination-monitoring controls. Third, host-DNA normalization was not performed, and microbial measurements were therefore analyzed as per-reaction copy numbers rather than normalized values per human genome equivalent or tissue input. Fourth, the study used a commercial targeted qPCR panel (Colonoflor Premium kit (Alpha-Lab, Russia)) originally designed for intestinal microbiota profiling and applied it here in an exploratory manner to FFPE tumor tissue. Analytical performance characteristics specific to the present FFPE application of the commercial panel, including per-target standard curves, amplification efficiency, and LOD/LOQ, were not independently evaluated within this study. In addition, the commercial user documentation did not disclose primer oligonucleotide sequences, which limited the extent to which assay design details could be reported in the manuscript.

Accordingly, the findings should be interpreted as exploratory associations of tumor-associated microbial DNA rather than as a fully matrix-validated characterization of the intratumoral microbiome.

## Data Availability

The raw data supporting the conclusions of this article will be made available by the authors, without undue reservation.

## Glossary

16S rRNA: 16S ribosomal RNA (gene)
A260/A280: absorbance ratio at 260/280 nm (spectrophotometric DNA purity metric)
AJCC: American Joint Committee on Cancer
ApcMin/+: adenomatous polyposis coli multiple intestinal neoplasia allele (mouse model genotype)
AUC: area under the curve
BFGS: Broyden–Fletcher–Goldfarb–Shanno algorithm
BH: Benjamini–Hochberg procedure
CI: confidence interval
CLR: centered log-ratio
CRC: colorectal cancer
DNA: deoxyribonucleic acid
FDR: false discovery rate
FFPE: formalin-fixed, paraffin-embedded
G1: grade 1 (well differentiated)
G2: grade 2 (moderately differentiated)
G3: grade 3 (poorly differentiated)
HC1: heteroscedasticity-consistent robust standard errors (HC1)
IQR: interquartile range
L1: L1 regularization (L1 penalty)
L2: L2 regularization (L2 penalty)
LASSO: least absolute shrinkage and selection operator
M: distant metastasis (TNM component)
N: regional lymph nodes (TNM component)
OR: odds ratio
PCR: polymerase chain reaction
PCoA: principal coordinates analysis
PERMANOVA: permutational multivariate analysis of variance
PERMDISP: permutational analysis of multivariate dispersions
qPCR: quantitative polymerase chain reaction
ROC: receiver operating characteristic
ROC-AUC: area under the receiver operating characteristic curve
rRNA: ribosomal RNA
Rx2: contingency table with R rows and 2 columns
RxC: contingency table with R rows and C columns
SD: standard deviation
SE: standard error
T: primary tumor (TNM component)
TE: Tris–EDTA buffer
TNM: Tumor–Node–Metastasis staging system
WHO: World Health Organization
χ²: chi-square
